# Complete mitochondrial genome and the phylogenetic position of the tigertooth croaker *Otolithes ruber* (Perciformes: Sciaenidae) 

**DOI:** 10.1080/23802359.2016.1247676

**Published:** 2017-02-23

**Authors:** Chang-Chang Guo, Min Liu, Jian-Jie Lin, Fang-Qun Dai

**Affiliations:** a Marine Biodiversity and Global Change Research Center, College of Ocean and Earth Sciences, Xiamen University, Xiamen City, Fujian Province, China;; b Fuzhou Marine and Fisheries Technology Center, Fuzhou City, Fujian Province, China;; c Yellow Sea Fisheries Research Institute, Chinese Academy of Fishery Science, Qingdao City, Shandong Province, China

**Keywords:** Bayesian tree, mitogenome, *Otolithes*, Perciformes, phylogenetic relationship, Sciaenidae

## Abstract

In this study, the complete mitogenome of the tigertooth croaker *Otolithes ruber* was first determined. This mitogenome is 16,589 bp in length, and consists of 37 genes with the typical gene order and direction of transcription in vertebrates. The overall nucleotide composition is: 27.4% A; 29.1% C; 16.1% G and 27.4% T. Sizes of the 22 tRNA genes range from 66 to 74 bp. Four start codons (ACG, CTG, GTG and ATG) and three stop (AGA, TAG and TAA/TA/T) codons were detected in 13 protein-coding genes. In the Bayesian treebased on the complete mitogenomes of 18 species (including *O. ruber*) from the family Sciaenidae, all nodes were strongly supported. The phylogenetic results suggested that *O. ruber* was closed to the black-spotted croaker *Protonibea diacanthus*.

The family Sciaenidae (Perciformes) is commonly known as croakers and drums. As an economically important group of fishes, it comprises about 270 species in 70 genera in the world (Nelson 2006). The tigertooth croaker *Otolithes ruber* is an widespread sciaenid species inhabiting shallow coastal waters down to 40 m in the Indian and West Pacific Oceans, west to South Africa, east to southern China and Queensland, Australia (Sasaki 2001). The species feeds mainly on fishes and prawns with maximum total length of 90 cm (http://www.fishbase.org). In this study, we presented the complete mitochondrial genome of *O. ruber* and assessed its phylogenetic relationship based on another 17 available mitogenomes in the family Sciaenidae with 2 available mitogenomes in the family Epinephelidae as an outgroup.

One specimen of *O. ruber* (MJ2015081503) was collected by a bottom trawler in the Min River Estuary, Fujian Province, China. The protocol and data analysis methods followed Chen et al. (2014). The complete mitochondrial genome of *O. ruber* is 16,589 bp in length (GenBank accession number: KX929060) with the typical gene order and transcriptional direction in vertebrates. It contains two rRNA genes, 22 tR 45 NA genes, 13 protein-coding genes and one control region. The overall nucleotide composition is as follows: 27.4% A, 29.1% C, 16.1% G and 27.4% T. In the 13 identified protein-coding genes, four start codons (ACG, 48 CTG, GTG and ATG) were detected; *ND1* was initiated by the ACG codon, *ATP8* by the GTG codon, *ATP6* by the CTG codon and the other 10 genes by the ATG codon. Three stop codons (AGA, TAG and TAA/TA/A) were found; *COX1* was terminated by the AGA codon, *ND1* by the TAG codon and the other 11 protein-coding genes by either the TAA or incomplete T or TA codon that may form complete termination signal UAA via post-transcriptional polyadenylation (Ojala et al. 1981). The 12S (958 bp) and 16S (1752 bp) rRNA genes are located between the tRNA-*Phe* and tRNA-*Leu1* genes, separated by the tRNA-*Val* gene. The lengths of 22 tRNA genes range from 66 to 74 bp; 21 tRNAs can be folded into the typical cloverleaf secondary structures with the exception of tRNA-*Ser2* in which the DHU arm was replaced by a simple loop. A 40 bp inserted sequence was identified as the putative origin of L-strand replication (OL). The control region was 838 bp in length with high A + T (64.5%) and low G + C (35.5%) composition.

Published mitogenomes of all 18 species of the family Sciaenidae (including *O. ruber* in this study), and the chocolate hind *Cephalopholis boenak* and the Hong Kong grouper *Epinephelus akaara* from the family Epinephelidae were used to assess the phylogenetic relationship of *O. ruber.* Phylogenetic tree was constructed with the partitioned Bayesian method based the dataset combined by three partitions (the alignments of the 1, 2 codon positions of 12 H-strand encoded protein-coding genes together with 2 rRNAs) under the GRT + I + G model (Ronquist & Huelsenbeck 2003). As the phylogenetic tree showed, all nodes were strongly supported with high value 67 of posterior probability (Figure 1). The result suggested that *O. ruber* was placed closely to *Protonibea diacanthus*, and subsequently to the group with genera *Pennahia*, *Nibea*, *Chrysochir*, *Dendrophysa* and *Johnius*. The resulting relationships are not consistent with the earlier conclusions based on the morphological characters. The genera *Otolithes*, *Chrysochir*, *Sonorolux*, *Larimichthys* and *Collichthys* formed a monophyletic group by having the distal end of the sulcus tail circular in their otolith (Sasaki 1989). It was also suggested that *Otolithes* and *Chrysochir* had a single synapomorphy, canines present (Sasaki 1989). A recent study based on mitochondrial (*Cyt b*, *COX1*) and nuclear (exon 3 of *RAG1*, *RH*, exon 2 of *EGR1* and exon 1, intron 1 and exon 2 of *EGR2B*) genes revealed that *Otolithes*, *Pterotolithus* and *Atrobucca* formed a monophyletic group which closed to a large group contained *Chrysochir*, *Protonibea*, *Megalonibea*, *Pennahia*, *Nibea*, *Dendrophysa*, *Austronibea*, *Daysciaena* and *Johnius* (Lo et al. 2015). Similar phylogenetic relationships in the genera of sciaenids were noted based on molecular analyses (Lo et al. 2015; this study). Therefore, more mitogenomes are needed to fully elucidate the evolution of *O. ruber* and the phylogenetic relationship of the sciaenid species.

**Figure 1.**
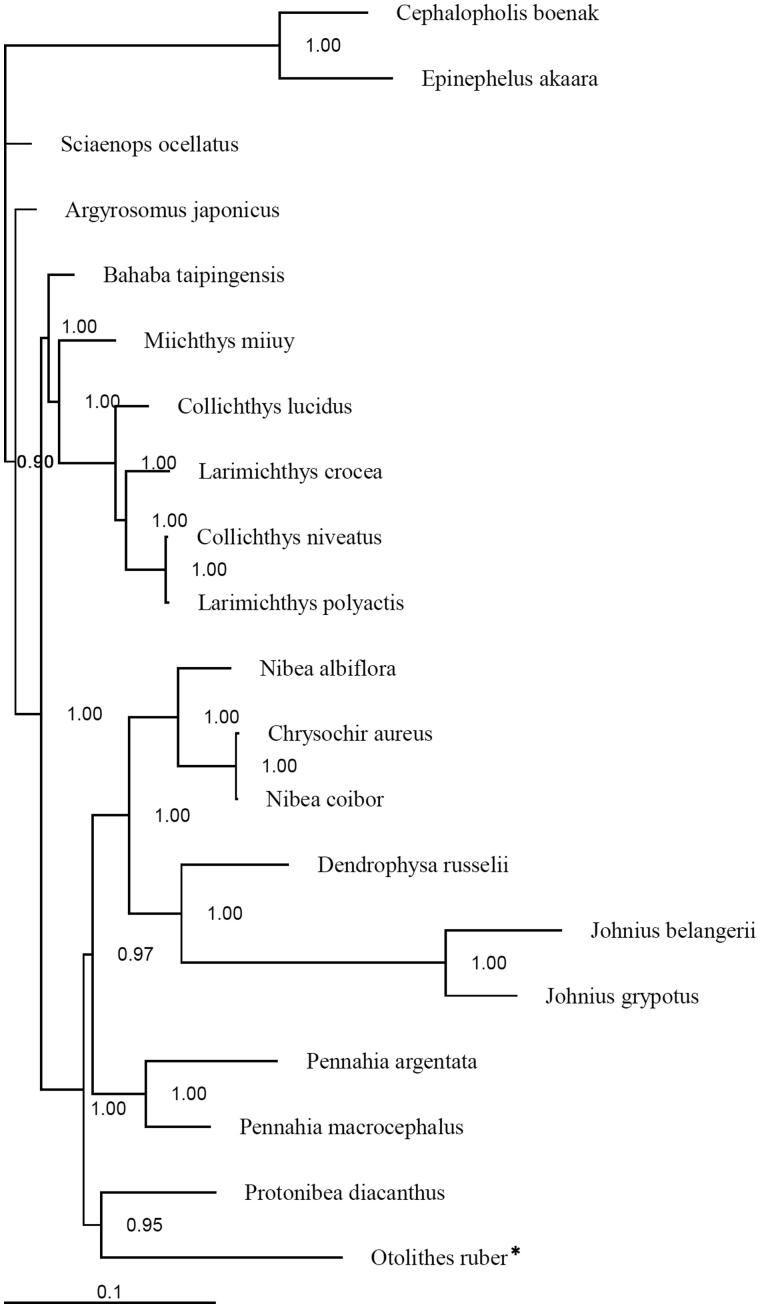
Phylogenetic positon of the tigertooth croaker *Otolithes ruber*.
